# DNA methylation biomarkers for hepatocellular carcinoma

**DOI:** 10.1186/s12935-018-0629-5

**Published:** 2018-09-17

**Authors:** Guorun Fan, Yaqin Tu, Cai Chen, Haiying Sun, Chidan Wan, Xiong Cai

**Affiliations:** 1 Department of Otorhinolaryngology, Union Hospital, Tongji Medical College, Huazhong University of Science and Technology, Wuhan, 430022 China; 2 Department of Hepatobiliary Surgery, Union Hospital, Tongji Medical College, Huazhong University of Science and Technology, Wuhan, 430022 China; 3 Department of Endocrinology, The Central Hospital of Wuhan, Tongji Medical College, Huazhong University of Science and Technology, Wuhan, China

**Keywords:** Hepatocellular carcinoma, Methylation, Hub genes

## Abstract

**Background:**

Aberrant methylation of DNA is a key driver of hepatocellular carcinoma (HCC). In this study, we sought to integrate four cohorts profile datasets to identify such abnormally methylated genes and pathways associated with HCC.

**Methods:**

To this end, we downloaded microarray datasets examining gene expression (GSE84402, GSE46408) and gene methylation (GSE73003, GSE57956) from the GEO database. Abnormally methylated differentially expressed genes (DEGs) were sorted and pathways were analyzed. The String database was then used to perform enrichment and functional analysis of identified pathways and genes. Cytoscape software was used to create a protein–protein interaction network, and MCODE was used for module analysis. Finally, overall survival analysis of hub genes was performed by the OncoLnc online tool.

**Results:**

In total, we identified 19 hypomethylated highly expressed genes and 14 hypermethylated lowly expressed genes at the screening step, and finally found six mostly changed hub genes including *MAD2L1*, *CDC20*, *CCNB1*, *CCND1*, *AR* and *ESR1*. Pathway analysis showed that aberrantly methylated-DEGs mainly associated with the cell cycle process, p53 signaling, and MAPK signaling in HCC. After validation in TCGA database, the methylation and expression status of hub genes was significantly altered and same with our results. Patients with high expression of *MAD2L1*, *CDC20* and *CCNB1* and low expression of *CCND1*, *AR*, and *ESR1* was associated with shorter overall survival.

**Conclusions:**

Taken together, we have identified novel aberrantly methylated genes and pathways linked to HCC, potentially offering novel insights into the molecular mechanisms governing HCC progression and serving as novel biomarkers for precision diagnosis and disease treatment.

**Electronic supplementary material:**

The online version of this article (10.1186/s12935-018-0629-5) contains supplementary material, which is available to authorized users.

## Background

Hepatocellular carcinoma (HCC), an inflammation-driven disease, is the third deadliest cancer worldwide, and HCC prevalence is predicted to continue to rise in coming years, serving as a major economic burden [1, 2]. Most cases of HCC occur in developing countries, such as China, and the leading cause of HCC is infection with hepatitis B virus (HBV); in contrast, the main cause in developed countries, such as the USA, is infection with hepatitis C virus (HCV) [3, 4]. Other risk factors for developing HCC include exposure to aflatoxin, alcohol intake, smoking, and diabetes [5]. The best curative treatments to date in early stage HCC patients involve surgical resection, tumor ablation, and potentially liver transplantation [6, 7]. However, the prognosis after curative therapy for HCC remains unsatisfactory because of a high postoperative recurrence rate. An improved understanding of the basic biology of HCC is needed to enhance prognostic predictions and to enhance therapeutic efficacy against this deadly disease.

The term epigenetics refers to heritable gene expression alterations no associated with DNA sequence changes [8]. The DNA methylation is closely related to embryonic development [9], regulation of gene expression [10], X-chromosome inactivation [11], genomic imprinting [12], and genomic stability [13]. Altered DNA methylation such as tumor suppressor gene hypermethylation or oncogene hypomethylation is thought to promote tumorigenesis. Genes including *P15*, *P16*, *Ras association domain family 1 isoform A* (*RASSF1A*), and *Retinoblastoma 1* are inactivated in HCC due to promoter hypermethylation of these genes [14–17]. Given that methylation is potentially reversible, detection of such aberrant DNA methylation of tumor suppressors and oncogenes in HCC could be useful as a therapeutic target.

While altered methylation of many genes has been demonstrated to date in the context of HCC, a complete interaction network documenting the relationship between said genes remains to be produced. The comprehensive analysis of multiple datasets offers the power needed to properly identify and assess pertinent pathways and genes mediating the biological processes associated with HCC. To this end, we used datasets from microarrays examining gene expression (GSE84402, GSE46408) and gene methylation (GSE73003, GSE57956) to assess HCC gene and epigenetic signatures, allowing for identification of genes and pathways that were both abnormally methylated and differentially expressed. Using a protein–protein interaction network we were also able to identify key so-called “hub” genes central to these signaling events. Through this analysis, we believe it is possible to identify novel differentially methylated genes associated with HCC, offering key insights into the molecular mechanisms governing HCC development and progression.

## Methods

### Microarray data

Gene expression profiling datasets GSE84402 and GSE46408, and gene methylation profiling datasets GSE73003 and GSE57956, were downloaded from the gene expression omnibus (GEO, https://www.ncbi.nlm.nih.gov/geo/) [18, 19]. 14 HCC and 14 normal specimens were obtained in GSE84402, while 6 HCC and 6 normal samples were obtained in GSE46408. GSE57956 included a total of 59 primary HCC tumor samples and 59 adjacent normal tissue samples, while GSE73003 included 40 paired normal and HCC samples from 20 patients.

### Data acquisition and processing

Raw gene expression profiling datasets GSE84402 and GSE46408 were downloaded from GEO public repositories. Data processing was performed using robust multi-array average (RMA) in GeneSpring GX 11.5 (Agilent Technologies Pty Ltd), including background adjustment, normalization and log transformation of the values. The threshold set for up and down-regulated genes was a | log FC | ≥ 2 and *P *≤ 0.05. For gene methylation profiling dataset (GSE73003 and GSE57956), the GEO2R software was used to analyze the raw data and to identify differentially methylated genes (DMGs). GEO2R is an interactive web tool which allows users to compare different groups of samples in a GEO series to screen genes that are differentially expressed in experimental conditions. The adjusted P-values (adj. P) and Benjamini and Hochberg false discovery rate were applied to provide a balance between discovery of statistically significant genes and limitations of false-positives. The DMGs cutoff criteria were *P* < 0.05 and |t| > 2. Additionally, one-sided tests were used to categorize the upregulated or downregulated DMGs. Finally, a Venn diagram was used to identify hypomethylated highly-expressed genes and hypermethylated lowly-expressed genes.

### Functional and pathway enrichment analysis

Gene ontology (GO) analyses and Kyoto Encyclopedia of Genes and Genomes (KEGG) pathway enrichment analyses were conducted on identified genes with altered methylation/expression using the Search Tool for the Retrieval of Interacting Genes (STRING, https://string-db.org/). This allowed for protein–protein interaction (PPI) network generation and functional annotation of genes of interest. *P* < 0.05 was the threshold for statistical significance.

### Generation and analysis of a protein–protein interaction (PPI) network

In order to interpret the drivers of carcinogenesis in a meaningful manner, a functional PPI analysis was necessary. The STRING database allowed us to generate PPI networks for both hypomethylated/and highly-expressed genes as well as for hypermethylated and lowly-expressed genes. A 0.4 interaction score served as the cutoff prior to visualization. Modules were then screened using Molecular Complex Detection (MCODE) in the Cytoscape software package, with MCODE score > 4 and number of nodes > 5. The top 3 Hub genes were selected by CytoHubba app in Cytoscape software. To determine the expression pattern of six hub genes in HCC, we used the datasets in the Oncomine (https://www.oncomine.org) and UALCAN (http://ualcan.path.uab.edu/) database. Oncomine and UALCAN are an online database consisting of previously published and open-access microarray data. The analysis enables multiple comparisons of gene expression between different studies; the significance of the gene expression across the available studies was also taken into account [20, 21]. The results were filtered by selecting hepatocellular carcinoma vs. normal tissue.

### Hub gene validation

The Cancer Genome Atlas (TCGA) database has generated comprehensive, multi-dimensional maps of the key genomic changes in various types of cancers. MEXPRESS (http://mexpress.be/) is a data visualization tool designed for the easy visualization of TCGA expression, DNA methylation and clinical data, as well as the relationships between them. To confirm our results, we used the MEXPRESS to validate hypermethylation/low-expression hub genes and hypomethylation/high-expression hub genes in TCGA database. The probability of survival and significance were calculated using the OncoLnc database. OncoLnc (http://www.oncolnc.org/) is an online tool for interactively exploring survival correlations, containing survival data for 8647 patients from 21 cancer studies.

## Results

### Identification of abnormally methylated and differentially expressed genes in HCC

**Fig. 1:**
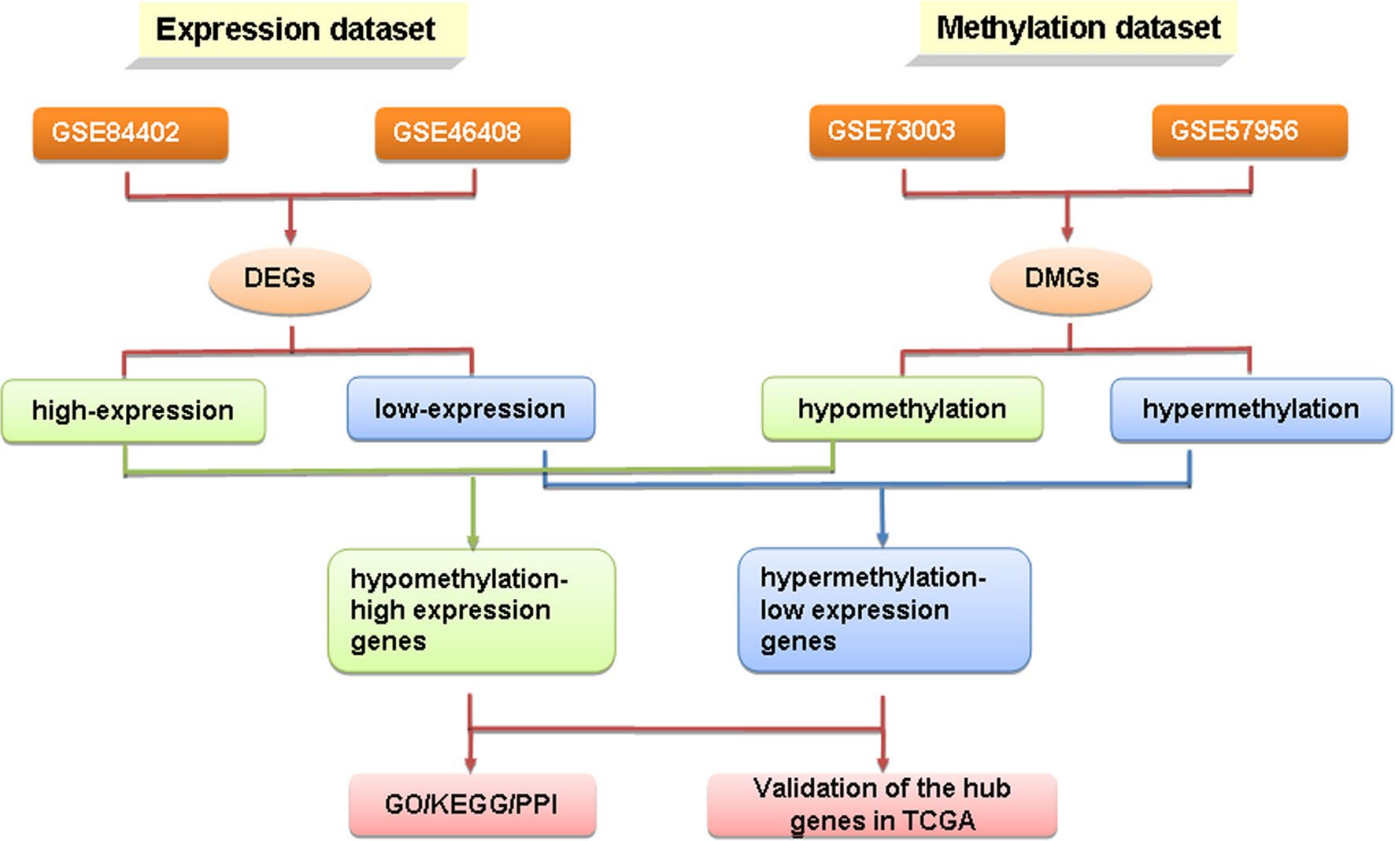
The flowchart of this study

**Fig. 2:**
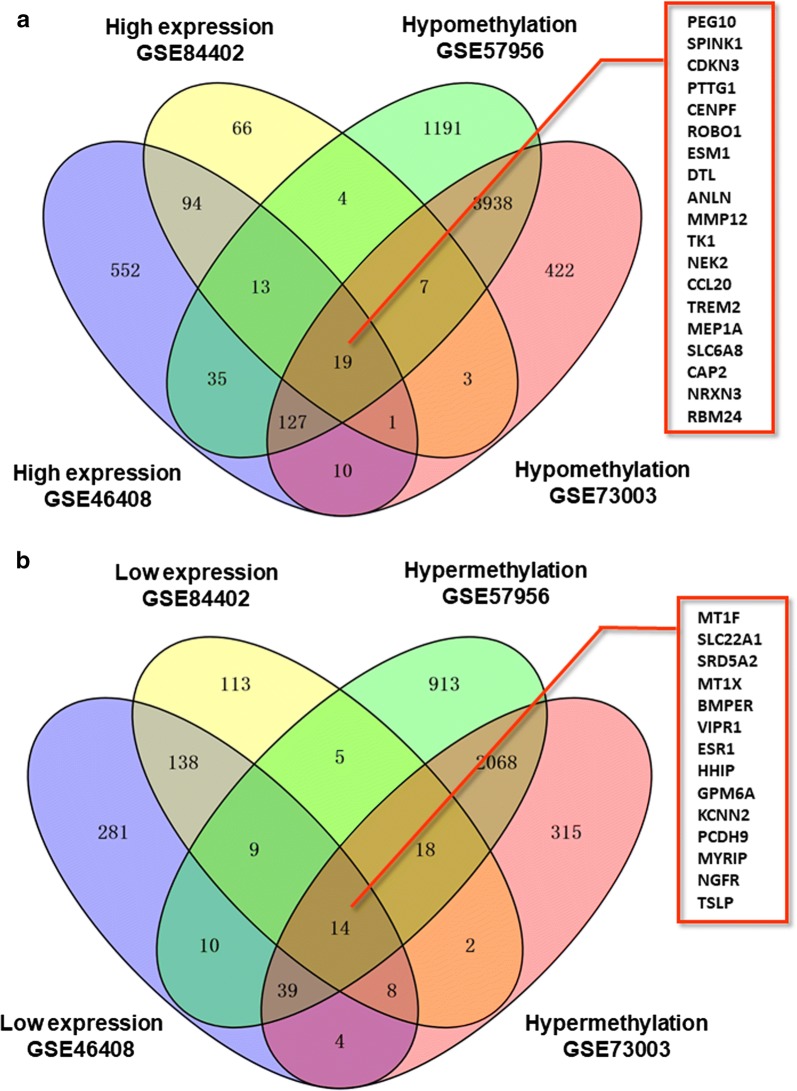
Identification of aberrantly methylated and differentially expressed genes was analyzed by Funrich software. Different color areas represented different datasets. **a** Hypomethylation and high expression genes; **b** hypermethylation and low expression genes

**Fig. 3:**
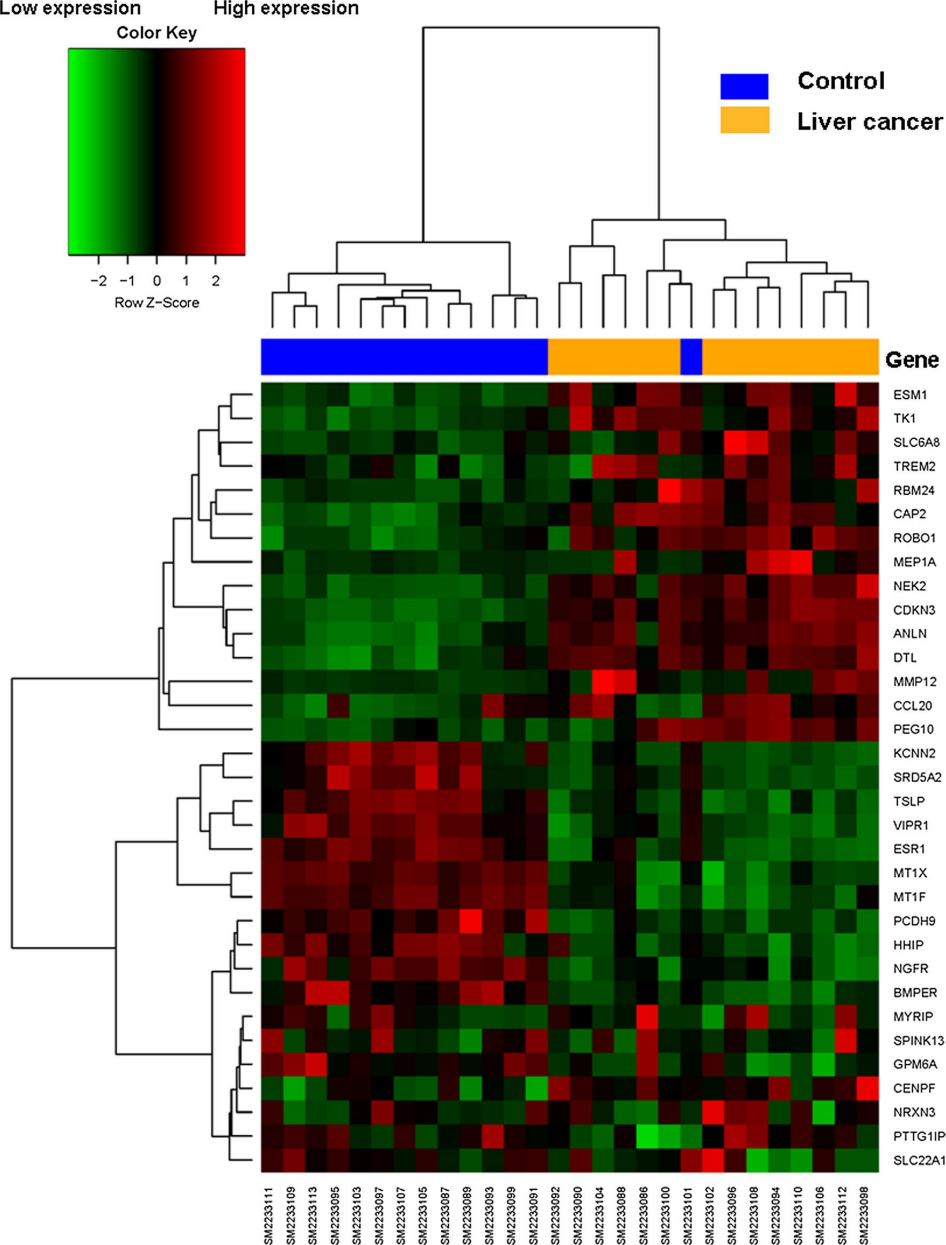
The heat map of 19 hypomethylation/high-expression genes and 14 hypermethylation/low-expression genes in GSE84402

A study design flowchart is shown in Fig. 1. In gene expression microarrays, 127 genes were up-regulated in both datasets (207 in GSE84402, 851 in GSE46408) and 169 were down-regulated in both datasets (307 in GSE84402, 503 in GSE46408). For the gene methylation microarrays, 2139 overlapping hypermethylated genes (2468 in GSE73003, 3157 in GSE57956) and 4091 overlapping hypomethylated genes (4527 in GSE73003, 5334 in GSE57956) were identified. By comparing these two sets of genes, a total of 19 hypomethylated, highly-expressed genes and 14 hypermethylated, low-expression genes were identified (Fig. 2). A heat map of these genes in GSE84402 is shown in Fig. 3.

### Gene ontology and pathway functional enrichment analysis

The GO annotation and pathway enrichment analyses of identified aberrantly methylated and differentially expressed genes were implemented using the online tool STRING. Genes that were hypomethylated and highly expressed were enriched for cell cycle regulation, while the hypermethylated low-expression genes were primarily linked to cell proliferation, gene expression, and signal transduction. Cell component enrichment analysis indicated that hypomethylated highly-expressed genes were correlated with cytoskeletal part, whereas hypermethylation/low-expression genes were predominant at intracellular organelle lumen. As for molecular function, hypomethylation/high-expression genes were enriched mainly in roundabout binding, histone kinase activity, and protein binding, while hypermethylation/low-expression genes were mostly enriched in cyclin binding, receptor binding, and signal transducer activity (Table 1). The pathway analysis revealed that hypomethylated highly-expressed genes were linked to oocyte meiosis, cell cycle, and ubiquitin mediated proteolysis, while hypermethylation/low-expression genes significantly enriched in pathways in cancer, p53 signaling pathway, MAPK signaling pathway, and proteoglycans in cancer (Table 2).

**Table 1:** Gene ontology analysis of aberrantly methylated-differentially expressed genes in hepatocellular carcinoma

Category	ID	Term	Gene count	False discovery rate
Hypomethylation and high expression				
BP	GO.0051983	Regulation of chromosome segregation	13	3.94E−20
BP	GO.0030071	Regulation of mitotic metaphase/anaphase transition	12	4.44E−20
BP	GO.0007094	Mitotic spindle assembly checkpoint	11	1.30E−19
BP	GO.0007088	Regulation of mitotic nuclear division	14	3.88E−19
BP	GO.0010564	Regulation of cell cycle process	17	2.77E−15
CC	GO.0000793	Condensed chromosome	10	3.89E−10
CC	GO.0005819	Spindle	11	6.30E−10
CC	GO.0031461	Cullin-RING ubiquitin ligase complex	7	1.34E−07
CC	GO.0005813	Centrosome	8	0.00011
CC	GO.0044430	Cytoskeletal part	12	0.000143
MF	GO.0048495	Roundabout binding	3	0.000105
MF	GO.0035173	Histone kinase activity	3	0.0059
MF	GO.0005515	protein binding	21	0.0083
Hypermethylation and low expression				
BP	GO.0042127	Regulation of cell proliferation	15	1.97E−06
BP	GO.0010468	Regulation of gene expression	19	0.000139
BP	GO.0009968	Negative regulation of signal transduction	10	0.000642
BP	GO.0000082	G1/S transition of mitotic cell cycle	5	0.00149
BP	GO.0051726	Regulation of cell cycle	9	0.00149
CC	GO.0097458	Neuron part	10	0.00143
CC	GO.0045202	Synapse	7	0.00802
CC	GO.0070013	Intracellular organelle lumen	16	0.0137
CC	GO.0005694	Chromosome	7	0.0138
CC	GO.0005654	Nucleoplasm	13	0.0149
MF	GO.0030332	Cyclin binding	4	4.14E−05
MF	GO.0005515	Protein binding	21	0.000265
MF	GO.0004871	Signal transducer activity	11	0.00462
MF	GO.0005102	Receptor binding	10	0.00462
MF	GO.0016538	Cyclin-dependent protein serine/threonine kinase regulator activity	3	0.00462

*BP* biological process, *CC* cell component, *MF* molecular function

**Table 2:** KEGG pathway analysis of aberrantly methylated-differentially expressed genes in hepatocellular carcinoma

Pathway ID	Pathway name	Gene count	False discovery rate	Genes
Hypomethylation and high expression				
4110	Cell cycle	12	1.03E−15	ANAPC10, ANAPC4, BUB1, BUB1B, BUB3, CCNB1, CDC16, CDC20, CDC23, MAD1L1, MAD2L1, PTTG1
4114	Oocyte meiosis	10	6.60E−13	ANAPC10, ANAPC4, AURKA, BUB1, CCNB1, CDC16, CDC20, CDC23, MAD2L1, PTTG1
4914	Progesterone-mediated oocyte maturation	8	1.84E−10	ANAPC10, ANAPC4, BUB1, CCNB1, CDC16, CDC23, MAD1L1, MAD2L1
4120	Ubiquitin mediated proteolysis	6	1.11E−05	ANAPC10, ANAPC4, CDC16, CDC20, CDC23, DDB1
4360	Axon guidance	4	0.00416	ROBO1, SLIT1, SLIT2, SLIT3
Hypermethylation and low expression				
5200	Pathways in cancer	9	3.99E−07	AR, CCND1, CDK4, CDK6, CDKN1A, HHIP, NTRK1, PTCH1, SHH
4115	p53 signaling pathway	4	0.000145	CCND1, CDK4, CDK6, CDKN1A
4110	Cell cycle	4	0.00109	CCND1, CDK4, CDK6, CDKN1A
4010	MAPK signaling pathway	5	0.00115	BDNF, NGF, NTF4, NTRK1, NTRK2
5205	Proteoglycans in cancer	4	0.00503	CCND1, CDKN1A, ESR1, PTCH1

### Construction and analysis of PPI networks

**Fig. 4:**
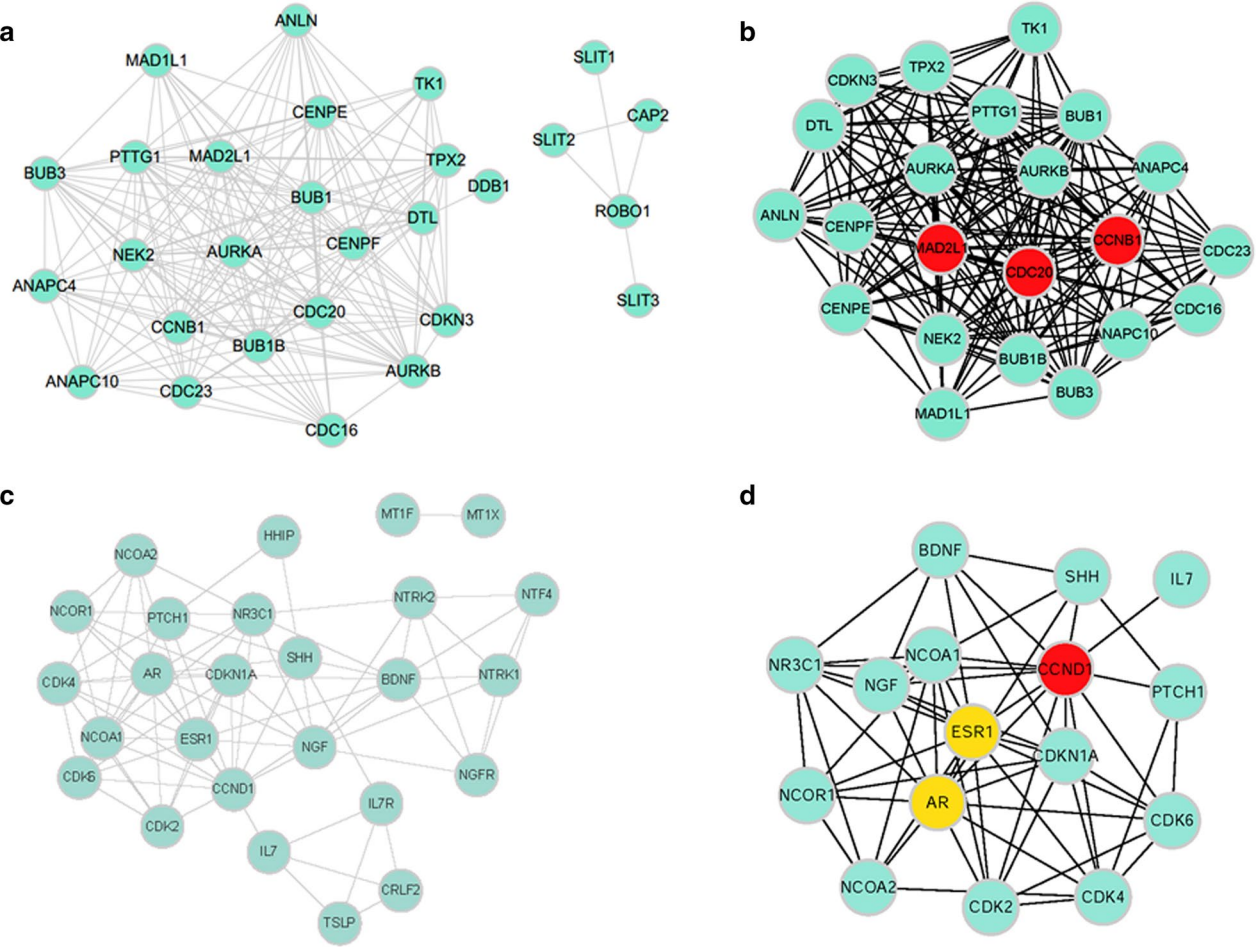
PPI network and hub genes of aberrantly methylated and differentially expressed genes. **a** PPI network and **b** Hub genes for hypomethylation/high-expression genes; **c** PPI network and **d** hub genes of hypermethylation/low-expression genes

**Fig. 5:**
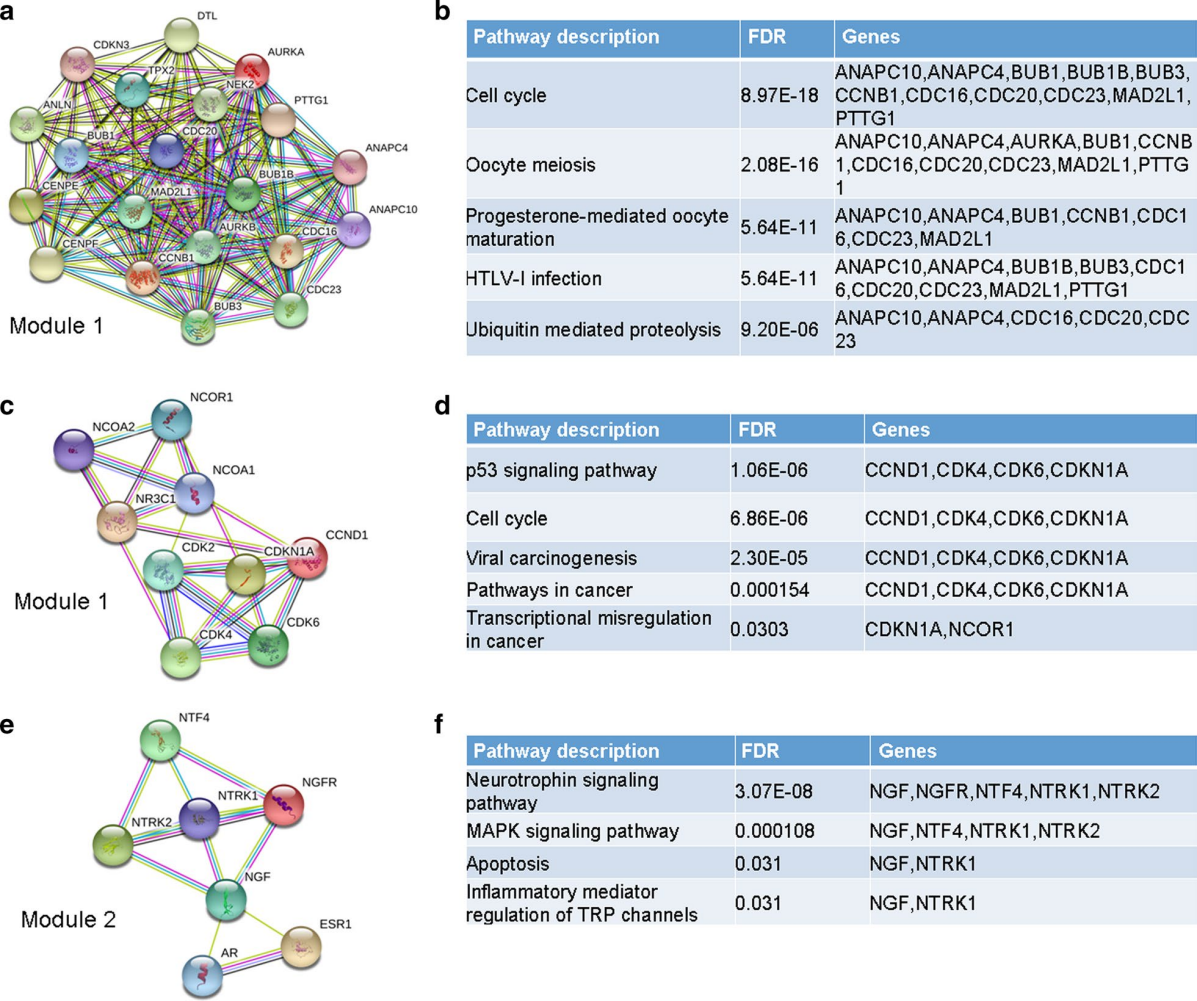
Core modules for aberrantly methylated and differentially expressed genes. Hypomethylation/high-expression genes **a** Module 1 and **b** the enrichment and pathways analysis of module 1; Hypermethylation/low-expression genes: **c** Module 1 and **d** the enrichment and pathways analysis of module 1; **e** Module 2 and **f** the enrichment and pathways analysis of module 2

**Fig. 6:**
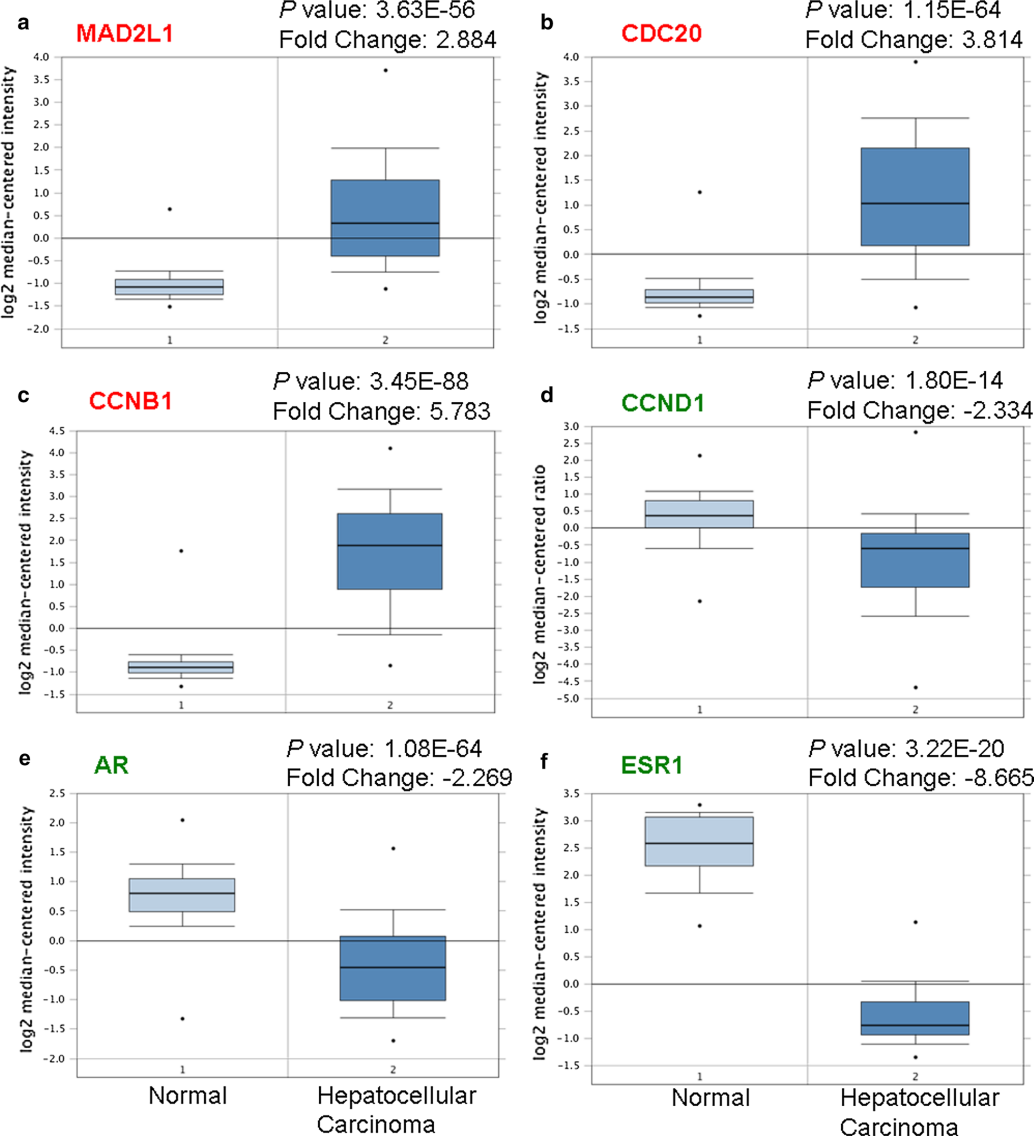
Validation of the expression of hub genes in Oncomine database. The expression level of **a** *MAD2L1*, **b** *CDC20*, **c** *CCNB1*, **d** *CCND1*, **e** *AR*, and **f** *ESR1* were detected in Oncomine database. Red: Hypomethylation/high-expression genes; Green: Hypermethylation/low-expression genes

The STRING database was used for PPI network construction, with MCODE used for module analysis. Hub genes were selected using the cytoHubba Cytoscape package. The PPI network for genes that were hypomethylated and highly expressed is shown in Fig. 4a, with corresponding modules shown in Fig. 5a. The most significantly enriched functional modules were those linked to progesterone-mediated oocyte maturation, the cell cycle, HTLV-I infection, oocyte meiosis, and ubiquitin mediated proteolysis (Fig. 5b). Top three hub genes were *MAD2L1*, *CDC20* and *CCNB1* (Fig. 4b). The PPI network for genes that were hypermethylated and expressed at low levels is shown in Fig. 4c, with corresponding modules shown in Fig. 5c, e. Significant vital modules showed functions including p53 signaling pathway, cell cycle, viral carcinogenesis, transcriptional misregulation in cancer, neurotrophin signaling pathway, MAPK signaling pathway, apoptosis and inflammatory mediator regulation of TRP channels (Fig. 5d, f). Top three hub genes were *CCND1*, *AR* and *ESR1* (Fig. 4d). Furthermore, we use Oncomine and UALCAN database to confirm the expression of hub genes in HCC (Fig. 6, Additional file 1: Figure S1). The data are in agreement with our results.

### Hub gene validation

Hub genes for the Hypermethylated/low-expression and Hypomethylation/high-expression datasets were then validated using the TCGA database in order to affirm the validity of these findings. The MEXPRESS web tool allowed for easy and rapid assessment of the relationships between methylation and gene expression within TCGA at a single-gene level. By ordering samples based on gene expression levels, MEXPRESS makes it clear that hub gene expression was negatively correlated with methylation, and a Pearson correlation confirmed this finding. For the hypermethylation/low-expression hub genes, gene expression tended to be higher in normal samples than in tumor samples (Additional file 2: Figure S2). However, tumor samples tended to have higher expression than normal samples for hypomethylation/high-expression hub genes (Additional file 3: Figure S3). The outcomes are summarized in Table 3. Gene expression and methylation were altered and consistent with our findings, indicating that these results are reliable and reproducible.

**Table 3:** Validation of the hub genes in TCGA database

Hub gene	Methylation status	P value	Expression status	P value
Hypomethylation/high-expression				
MAD2L1	Hypomethylation	< 2.2e−16	High expression	< 2.2e−16
CDC20	Hypomethylation	6.12e−13	High expression	< 2.2e−16
CCNB1	Hypomethylation	2.87e−5	High expression	< 2.2e−16
Hypermethylation/low-expression				
CCND1	Hypermethylation	1.27e−5	Low expression	1.27e−5
AR	Hypermethylation	2.96e−4	Low expression	1.47e−9
ESR1	Hypermethylation	0.0258	Low expression	< 2.2e−16

### The relationship between hub genes and the survival in hepatocellular carcinoma

**Fig. 7:**
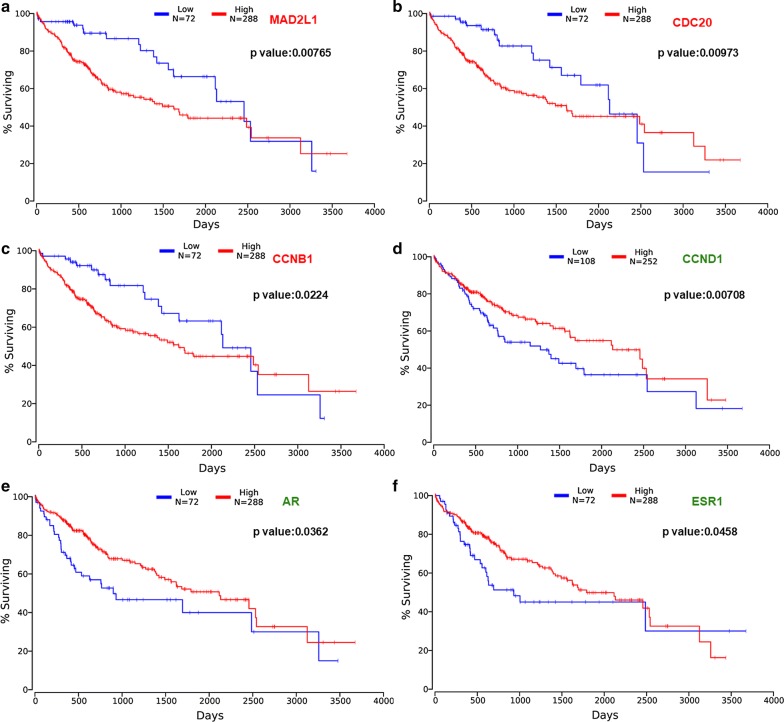
Prognostic value of six hub genes in hepatocellular carcinoma. Prognostic value of **a** *MAD2L1*, **b** *CDC20*, **c** *CCNB1*, **d** *CCND1*, **e** *AR*, and **f** *ESR1* were obtained in OncoLnc database. The survival curve comparing the patients with high (red) and low (blue) expression in HCC

The prognostic value of six hub genes was assessed by OncoLnc database. The threshold was adjusted to cox *P* value < 0.05. Patients with high expression of *MAD2L1*, *CDC20* and *CCNB1* and low expression of *CCND1*, *AR*, and *ESR1* was associated with shorter overall survival (Fig. 7). The survival data showed that *MAD2L1*, *CDC20* and *CCNB1* played an oncogenic role, while *CCND1*, *AR*, and *ESR1* genes were associated with better survival in HCC.

## Discussion

Exploring the mechanisms underlying HCC development and progression not only has prognostic implications, but may also be helpful in monitoring treatment response, surveillance for tumor recurrence, and guidance of clinical decisions. Modern advances in sequencing technologies and microarray development have provided ample high-throughput opportunities to study disease-related biology, allowing for simultaneous assessment of gene methylation and expression for thousands of genes in the human genome. In the present study, we identified 19 hypomethylated, highly-expressed genes and 14 hypermethylated, low-expression genes, using bioinformatic analysis. Functional enrichment of these genes revealed that aberrant methylation indeed affects certain pathways and hub genes. These results can provide novel insight into the explanation of HCC pathogenesis.

The GO enrichment analysis revealed that the primary biological processes of the hypomethylated/highly-expressed genes were the regulation of cell cycle processes, chromosome segregation, and mitotic nuclear division while the hypermethylated/low-expression genes were involved mainly in controlling cell proliferation, gene expression, and the mitotic G1/S transition. This is expected given that the chromosome segregation process occurs during mitosis, which is a part of the cell cycle. The G1/S cell cycle transition is tightly controlled. Deregulation of this key checkpoint can allow cells to undergo transformation, thereby permitting tumorigenesis. This finding is consistent with the fundamental role played by cell cycle regulators in cell proliferation, invasion, and metastasis in HCC. KEGG pathway analysis of hypomethylation/high-expression genes revealed that they were linked to the cell cycle, oocyte meiosis, and ubiquitin-mediated proteolysis. The cell cycle and oocyte meiosis are vital for cell proliferation in tumor cells, and the ubiquitin proteasome pathway functions to regulate cell cycle control and the DNA damage response in tumor genesis [22]. The KEGG pathway analysis of hypermethylation/low-expression genes suggested that methylation may be linked to HCC development through the p53 and MAPK signaling pathways. p53 is a tumor suppressor to conserve genome stability by preventing mutations caused by cellular stress or DNA damage. Together, these results suggest that hypermethylation and hypomethylation are key mediators of cancer development and progression.

The PPI network of hypomethylated/highly-expressed genes provides insight into the functional associations between them, and from this, the top three hub genes were selected: *MAD2L1*, *CDC20*, and *CCNB1*. *Mitotic Arrest Deficient 2 Like 1* (*MAD2L1*) and *Cell Division Cycle 20* (*CDC20*) are two key mitotic checkpoint genes. Both *MAD2L1* and *CDC20* were more highly expressed in higher grade tumors than in low-grade tumors. High *MAD2L1* or *CDC20* levels may allow for the development of multi-organ aggressive tumors, including those affecting the breasts, lungs, liver, and stomach [23–25]. Collectively, these findings suggest that MAD2L1 and CDC20 may be key regulators of tumor severity, ultimately dictating patient survival. *Cyclin B1 (CCNB1)*, complexing with CDC2, is a G2/M-phase checkpoint regulator that is vital for regulation of proliferation and DNA synthesis. CCNB1 overexpression has been found to occur in HCC [26] and many other cancer, often being linked to progression, recurrence, and to poor prognoses [27]. Therefore, *MAD2L1*, *CDC20*, and *CCNB1* may all be abnormally methylated genes that modulate the cell cycle and proliferation in HCC. With regard to the hypermethylated/low-expression genes, the most prominent hub genes were *CCND1*, *AR*, and *ESR1*. *Cyclin D1 (CCND1)* is a proto-oncogene regulating G1 to S phase progression; it participates in the Wnt/β-catenin pathway [28, 29]. *Androgen receptor* (*AR*) is a steroid hormone receptor superfamily member that is involved in human hepatocarcinogenesis [30]. It alters the AR-dependent transcriptome and stimulates oncogenic growth. *Estrogen receptor 1* (*ESR1*) functions as a transcription factor, regulating cell cycle, cell proliferation, apoptosis, and inflammation-associated gene expression [31]. Research has shown that estrogen-depleted postmenopausal women undergo more rapid progression from HCV-infection to HCC development [32]. Aberrant expression of *ESR* subtypes may contribute to the progression of HCC. These three genes are related to prognosis, tumorigenesis, and metastasis of HCC. Furthermore, survival analysis of hub genes revealed that *MAD2L1, CDC20 and CCNB1* play an oncogenic role, while *CCND1, AR, and ESR1* genes were associated with favorable patient survival in HCC.

The core PPI network module for hypomethylated/highly-expressed genes was linked to the cell cycle, oocyte meiosis, and ubiquitin-mediated proteolysis, indicating that these pathways are key targets of hypermethylation. The top two modules of the hypermethylated/low-expression gene PPI network were those linked to the p53 signaling pathways, viral carcinogenesis and neurotrophin signaling pathways. p53 signaling conserves the stability of the genome. The leading cause of HCC is infection with HBV or HCV. It is reasonable that viral carcinogenesis is involved in the development of HCC. Neurotrophins and neurotrophin receptors are found on tumor and stromal cells, and are linked to many kinds of tumor development. Brain-derived neurotrophic factor and nerve growth factor are both neurotrophins linked to tumor development, promoting proliferation, angiogenesis, and invasion.

While previously groups have assessed arrays cataloging gene expression or methylation, the two have not been examined simultaneously. Furthermore, single studies generally lack the power needed to identify critical regulatory genes and signaling pathways. Our research used a bioinformatics workflow to jointly analyze extant gene expression and gene methylation profiling microarrays, allowing for more powerful and precise insights into these screening results. However, we only validated candidate abnormally methylated genes that were differentially expressed using the TCGA database. Further experiments will be necessary in order to confirm that these genes and pathways are linked to HCC.

## Conclusion

In summary, this study provides a comprehensive bioinformatics analysis of aberrantly methylated DEGs that may be involved in the progression and development of HCC. In addition, six mostly changed hub genes were identified, including *MAD2L1*, *CDC20*, *CCNB1*, *CCND1*, *AR*, and *ESR1*. These novel findings may contribute to the unraveling of the pathogenesis of HCC, and these candidate genes may be optimal abnormal methylation-based biomarkers that can be used to accurately diagnose and treat HCC.

## Additional files

**Additional file 1: Figure S1.** Validation of the expression of hub genes in UALCAN database. Red: Hypomethylation/high-expression genes; Green: Hypermethylation/low-expression genes.

**Additional file 2: Figure S2.** Validation of the hypermethylation/low-expression hub genes in TCGA database. For the hypermethylation/low-expression hub genes, normal samples tended to have higher expression than tumor samples.

**Additional file 3: Figure S3.** Validation of the hypomethylation/high-expression hub genes in TCGA database. Tumor samples tended to have higher expression than normal samples for hypomethylation/high-expression hub genes.

## Abbreviations

HCC: hepatocellular carcinoma
DEGs: differentially expressed genes
DMGs: differentially methylated genes
PPI: protein-protein interaction
RASSF1A: Ras association domain family 1 isoform A
GEO: gene expression omnibus
KEGG: Kyoto Encyclopedia of Genes and Genomes
STRING: Search Tool for the Retrieval of Interacting Genes
MCODE: Molecular Complex Detection
TCGA: The Cancer Genome Atlas
GO: gene ontology
MAD2L1: Mitotic Arrest Deficient 2 Like 1
CDC20: Cell Division Cycle 20
CCNB1: cyclin B1
CCND1: cyclin D1
AR: androgen receptor
ESR1: estrogen receptor 1
NGF: nerve growth factor
BDNF: brain-derived neurotrophic factor
