# Supplementary figures and images for: DNA methylation biomarkers for hepatocellular carcinoma
Source: Cancer Cell Int. 2018 Sep 17;18:140. doi: 10.1186/s12935-018-0629-5 (PMC6142709; doi:10.1186/s12935-018-0629-5)

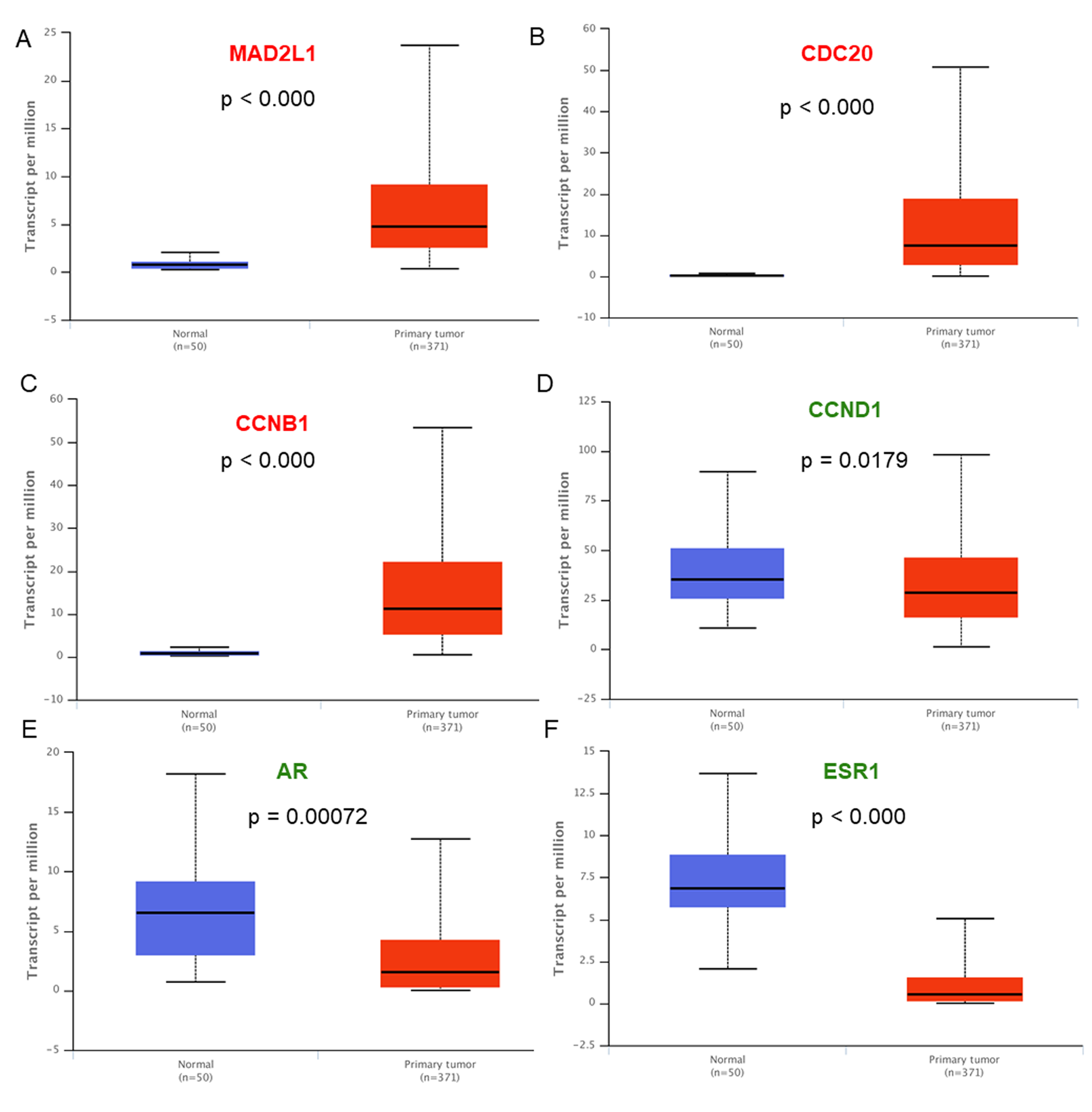

Supplement: Supplementary file 1 — Additional file 1: Figure S1. Validation of the expression of hub genes in UALCAN database. Red: Hypomethylation/high-expression genes; Green: Hypermethylation/low-expression genes. [file 12935_2018_629_MOESM1_ESM.tif]

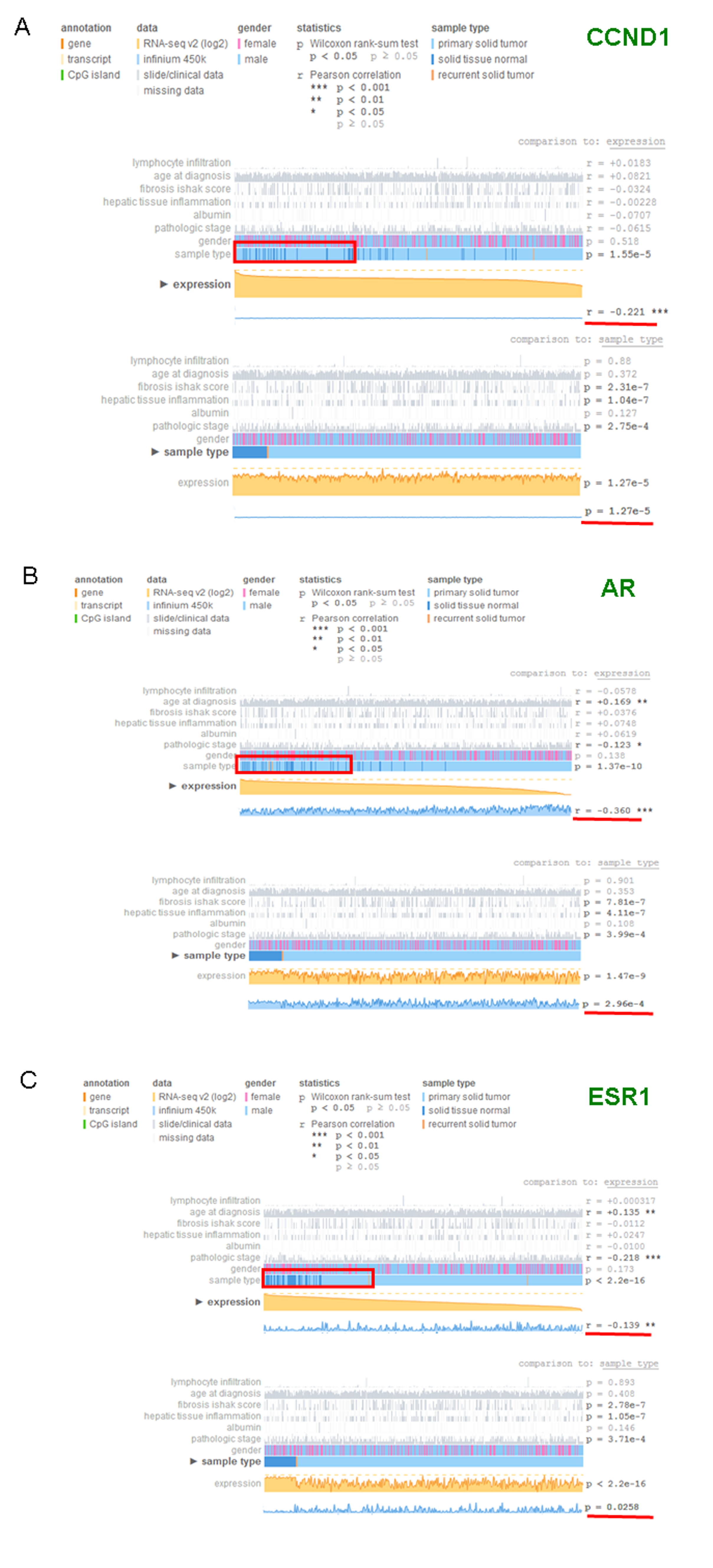

Supplement: Supplementary file 2 — Additional file 2: Figure S2. Validation of the hypermethylation/low-expression hub genes in TCGA database. For the hypermethylation/low-expression hub genes, normal samples tended to have higher expression than tumor samples. [file 12935_2018_629_MOESM2_ESM.tif]

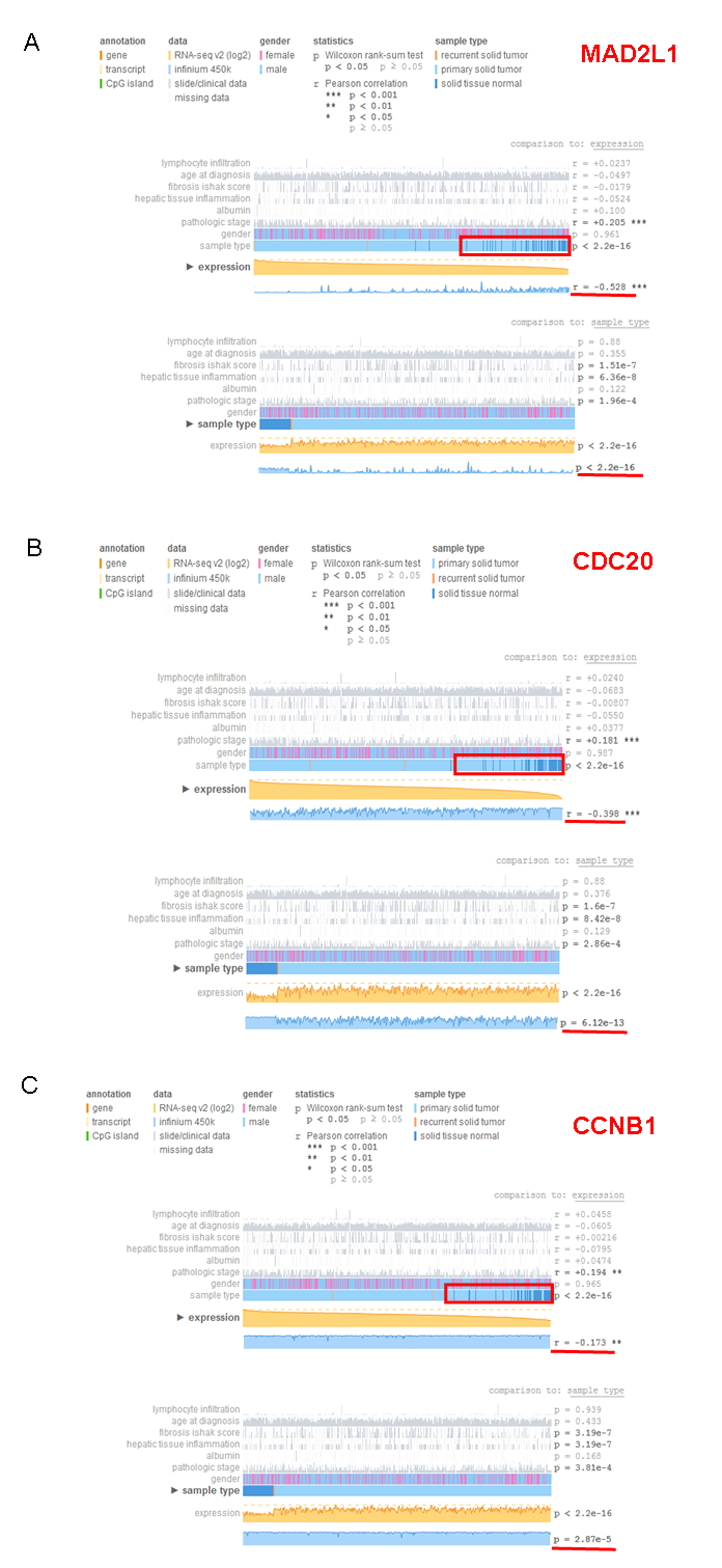

Supplement: Supplementary file 3 — Additional file 3: Figure S3. Validation of the hypomethylation/high-expression hub genes in TCGA database. Tumor samples tended to have higher expression than normal samples for hypomethylation/high-expression hub genes. [file 12935_2018_629_MOESM3_ESM.tif]
